# β-Hydroxybutyrate inhibits inflammasome activation to attenuate Alzheimer’s disease pathology

**DOI:** 10.1186/s12974-020-01948-5

**Published:** 2020-09-21

**Authors:** Daniel C. Shippy, Connor Wilhelm, Patel A. Viharkumar, Thomas J. Raife, Tyler K. Ulland

**Affiliations:** Department of Pathology and Laboratory Medicine, University of Wisconsin, Madison, WI USA

**Keywords:** Alzheimer’s disease, Neuroinflammation, β-Hydroxybutyrate, Inflammasome, Metabolism, Microglia

## Abstract

Alzheimer’s disease (AD) is a progressive, late-onset dementia with no effective treatment available. Recent studies suggest that AD pathology is driven by age-related changes in metabolism. Alterations in metabolism, such as placing patients on a ketogenic diet, can alter cognition by an unknown mechanism. One of the ketone bodies produced as a result of ketogenesis, β-hydroxybutyrate (BHB), is known to inhibit NLRP3 inflammasome activation. Therefore, we tested if BHB inhibition of the NLRP3 inflammasome reduces overall AD pathology in the 5XFAD mouse model of AD. Here, we find BHB levels are lower in red blood cells and brain parenchyma of AD patients when compared with non-AD controls. Furthermore, exogenous BHB administration reduced plaque formation, microgliosis, apoptosis-associated speck-like protein containing a caspase recruitment domain (Asc) speck formation, and caspase-1 activation in the 5XFAD mouse model of AD. Taken together, our findings demonstrate that BHB reduces AD pathology by inhibiting NLRP3 inflammasome activation. Additionally, our data suggest dietary or pharmacological approaches to increase BHB levels as promising therapeutic strategies for AD.

## Introduction

Alzheimer’s disease (AD) is a neurodegenerative disorder characterized by memory loss and impaired cognitive functions. Recent evidence suggests age-related metabolic dysfunction plays a role in promoting cognitive impairment and overall AD pathology [1–5]. Key features of AD pathology include the accumulation of amyloid-β (Aβ) plaques followed by the formation of neurofibrillary tangles (NFTs) [6]. In response to Aβ aggregation, microglia promote Aβ and tau clearance and create physical barriers to protect neurons from neurotoxic plaque, but also drive neuroinflammation that damages neurons [7–12]. Chronic activation of the nod-like receptor family pyrin domain containing 3 (NLRP3) inflammasome has emerged as an important mechanism in continual neuroinflammation that significantly increases AD pathology [13]. Therefore, inhibiting NLRP3 activation could be a promising therapeutic strategy for AD.

The ketogenic diet is a high-fat, low-carbohydrate dietary regimen that stimulates the breakdown of fatty acids and ketogenic amino acids, a process that results in the production of ketone bodies that can be further metabolized for energy production. The three ketone bodies are acetone, acetoacetate, and β-hydroxybutyrate (BHB). Previous work has shown that BHB is present at significantly lower levels in the blood of AD patients [14]. Furthermore, higher circulating BHB levels in the blood of AD patients are correlated with increased scores on cognitive tests [14–18], and BHB has been shown to modify disease in mouse models of AD by unknown mechanisms [19–21]. Recently, BHB was shown to inhibit NLRP3 inflammasome activation in human monocytes [22]. Given these findings, and that BHB can effectively cross the blood–brain barrier, further investigation is warranted to determine a potential mechanism by which BHB attenuates AD pathology.

In this study, we demonstrate that exogenous BHB administration to mice carrying human presenilin 1 (PSEN1) and amyloid precursor protein (APP) with 5 familial AD mutations under the CD90 promoter, which deposit Aβ plaques [23] (5XFAD mice), decreases overall AD pathology through inhibition of the inflammasome. We found that BHB levels in brain tissue and red blood cell samples from AD patients were significantly lower than those from non-AD controls. We then confirmed that BHB inhibits the NLRP3 inflammasome in bone marrow-derived macrophages (BMDM) by decreased levels of IL-1β and caspase-1. Using the 5XFAD mouse model of AD, BHB treatment resulted in dramatically fewer cortical plaques accompanied by decreased cortical volume occupied by plaques. We also observed less pronounced microgliosis in the BHB-treated 5XFAD mice, which had significantly less processed caspase-1 in their cortices. Consistent with a decrease in NLRP3 inflammasome activation, we observed significantly fewer apoptosis-associated speck-like protein containing a caspase recruitment domain (Asc) specks in the BHB-treated 5XFAD mice. Overall, our findings demonstrate that augmenting BHB levels may be a promising preventative and therapeutic strategy for AD.

## Results and discussion

### AD patients have lower levels of BHB in their blood and brain parenchyma

**Fig. 1:**
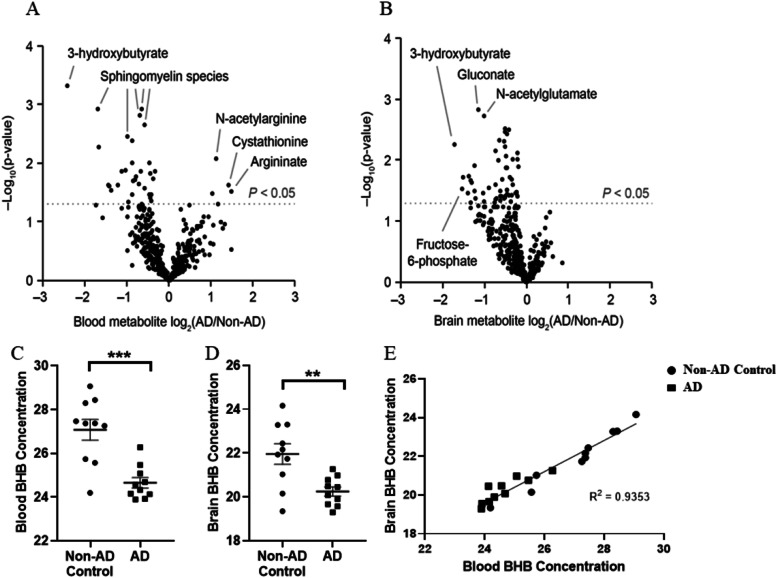
AD patients have less BHB in their blood and brain parenchyma. Volcano plots showing the relative concentration of various metabolites detected in the red blood cells (**a**) and brain parenchyma (**b**) of AD patients compared with non-AD controls (CTRL). Relative expression is on the *x*-axis with significance on the *y*-axis. Graphs showing relative concentration of BHB in blood (**c**) and brain parenchyma (**d**) of AD patients compared with non-AD CTRL. Data are shown as mean ± SEM with ***P ≤ 0.001 and **P ≤ 0.01. (**e**) Correlation of BHB concentration in the brain of AD and non-AD CTRL with blood of AD and non-AD CTRL showing a strong, positive correlation of BHB in the brain and blood, with an *R*^2^ value of 0.9353. All data represent results taken from AD patients (*n* = 10) and non-AD CTRL (*n* = 10)

Recent studies suggest AD pathology is driven by age-related metabolic dysfunction in the brain [1, 2]. Also, during the immune response to AD onset, the metabolic state of microglia affects disease progression [3–5]. Glucose appears to be the main energy source for both microglial and brain energy metabolism, but other substrates, like ketone bodies, can be used as an alternative energy source. Surprisingly, one of the three ketone bodies, BHB, has been shown to be present at significantly lower levels in the blood of AD patients [14]. In our study, we found BHB at significantly lower levels in the red blood cells of AD patients when compared with non-AD controls (*P* = 0.0002; two-tailed Student’s *t* test) (Fig. 1a, c). We also found that BHB levels were significantly reduced in the brain tissue of AD patients when compared with non-AD controls (*P* = 0.0036; two-tailed Student’s *t* test] (Fig. 1b, d) with a strong correlation between BHB levels in the brain and red blood cells (Fig. 1e). Previous work has demonstrated that higher circulating levels of BHB in AD patients are correlated with increased scores on cognitive tests [14–18]. The mechanism by which ketones, including BHB, improve cognition in AD patients is unclear. One of the most important known risk factors for developing AD is diabetes [24]. Several studies have demonstrated impaired insulin signaling and insulin resistance in the brain to AD pathogenesis [25–28]. A growing body of evidence suggests ketones can significantly improve glucose homeostasis which in turn reduces insulin resistance and metabolic dysfunction [29, 30]. Clinical symptoms of AD rarely appear before decreases in the cerebral metabolism of glucose, indicating a link to local brain insulin resistance [26]. Ketone body metabolism can overcome insulin resistance, suggesting a therapeutic potential for ketone bodies in AD [31, 32]. Our data indicate that BHB levels are lower in AD patients, suggesting an important role for BHB in limiting AD pathology.

### BHB treatment leads to fewer plaques in 5XFAD mice

**Fig. 2:**
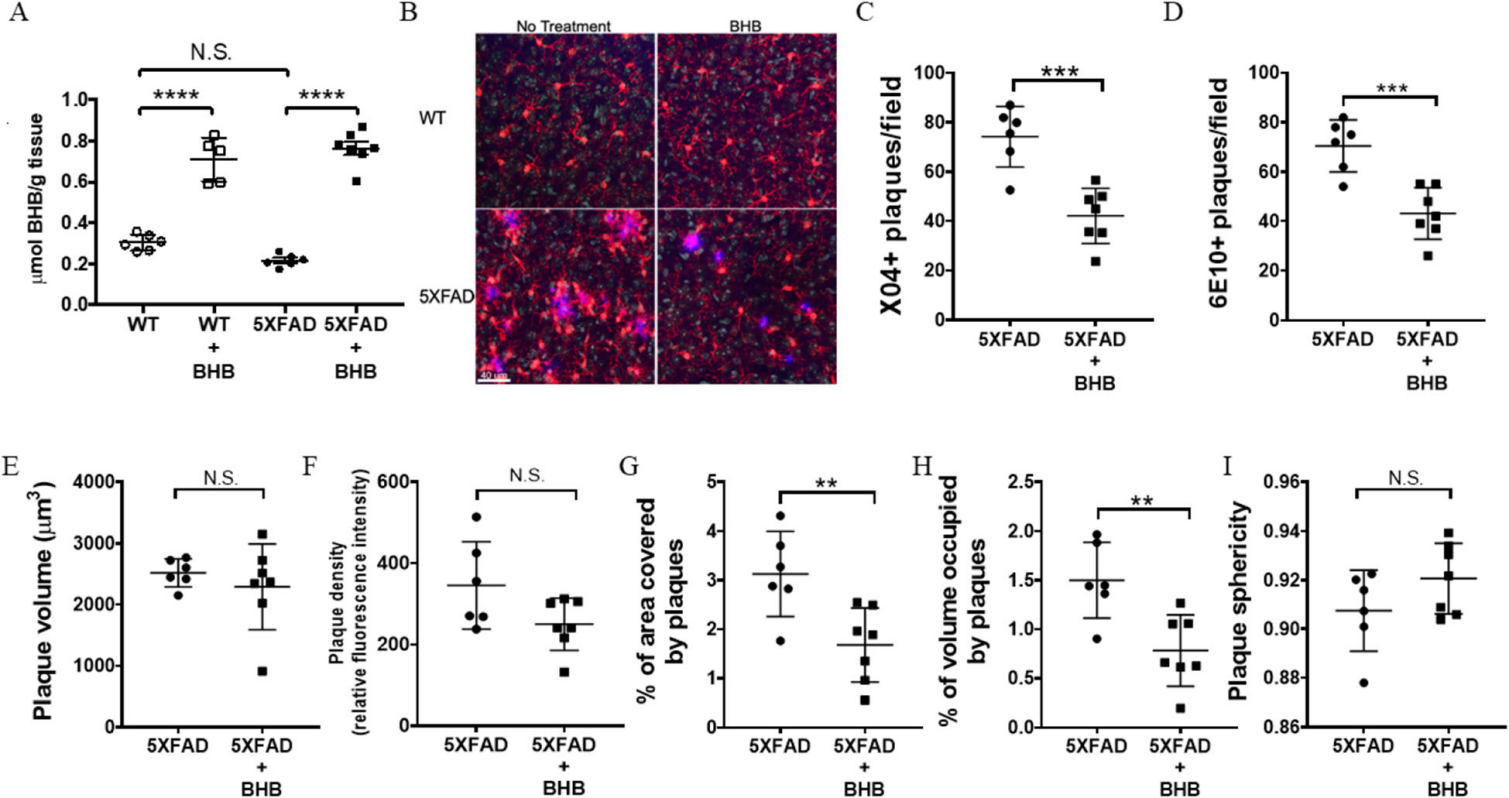
BHB treatment leads to fewer plaques in 5XFAD mice. (**a**) BHB levels in the cortices of mice (*n* = 5–7/group) as determined by colorimetric assay (Abcam). (**b**) Brain sections of WT and 5XFAD mice on regular water and BHB-containing water were stained for microglia (Iba1; red), nuclei (TO-PRO-3; cyan), and plaques (methoxy-X04; blue). Images were taken on a Nikon A1R confocal microscope. The number of X04+ plaques per field (**c**), number of 6E10+ plaques per field (**d**), plaque volume (**e**), plaque density (**f**), percent of area covered by plaques (**g**), percent of volume occupied by plaques (**h**), and plaque sphericity (**i**) were quantified using Imaris 9.2.1. Two to three images per mouse were quantified and averaged. The data shown were collected from 6–7 mice per group. Graphs show data as the mean ± SD with ****P ≤ 0.0001, ***P ≤ 0.001, **P ≤ 0.01, and N.S. = not significant

In order for BHB to be used as a therapeutic agent for AD, it must be capable of effectively crossing the blood–brain barrier. Endothelial cells of blood vessels in the brain express monocarboxylate transporters which mediate ketone body transport across the blood–brain barrier [33, 34]. In our study, BHB levels in the brain parenchyma of wild-type (WT) and 5XFAD mice were determined using a colorimetric ELISA. BHB levels in WT and 5XFAD control mice were minimal, with no significant difference between the groups. In contrast, WT and 5XFAD mice receiving BHB-treated water showed significantly increased levels of BHB compared with control mice receiving regular water (*P* ≤ 0.0001; one-way ANOVA with a Tukey’s multiple comparison test), indicating exogenous BHB administration in water allowed BHB to effectively cross the blood–brain barrier (Fig. 2a).

Aβ plaques are a hallmark of AD pathology [6]. Therefore, we compared plaque formation in the cortices of mice receiving untreated and BHB-treated water using confocal microscopy (Fig. 2b). 5XFAD mice receiving BHB-treated water had significantly fewer X04+ plaques (42.1 plaques/HPF) compared with untreated control mice (74.3 plaques/HPF) (*P* = 0.0005; two-tailed Student’s *t* test) (Fig. 2c). These findings were confirmed, as 5XFAD mice receiving BHB-treated water had significantly fewer 6E10+ plaques (43.1 plaques/HPF) compared with untreated control mice (70.5 plaques/HPF) (*P* = 0.0007; two-tailed Student’s *t* test) (Fig. 2d). Furthermore, 5XFAD BHB-treated mice showed a significantly reduced percent area covered by plaques (*P* = 0.0082; two-tailed Student’s *t* test) (Fig. 2g) and percent volume occupied by plaques (*P* = 0.0054; two-tailed Student’s *t* test) (Fig. 2h) when compared with 5XFAD control mice on untreated water. No significant differences were observed between BHB-treated and untreated mice for plaque volume (Fig. 2e), plaque density (Fig. 2f) or plaque sphericity (Fig. 2i). The mechanism in which BHB reduces Aβ plaque formation remains unclear. Several studies in AD mouse models show reduced tau and amyloid pathologies by unknown mechanisms when mice are placed on a ketogenic diet or administered BHB [19, 20, 35]. In cell culture models of AD, the addition of Aβ_1–42_ results in cell death caused by altered mitochondrial pyruvate activity, but can be rescued by the addition of BHB, which provides an alternative mitochondrial substrate when glucose is unavailable to be used as an energy source [31, 36, 37]. Additionally, the study by Yao and colleagues suggests dietary supplementation with 2-deoxy-d-glucose induces ketoacidosis, improves mitochondrial function, and attenuates Aβ pathology in a mouse model of AD [38]. These findings are validated by the observation that mitochondrial bioenergetics deficits are correlated with AD pathogenesis [39].

### BHB treatment decreases microgliosis in 5XFAD mice

**Fig. 3:**
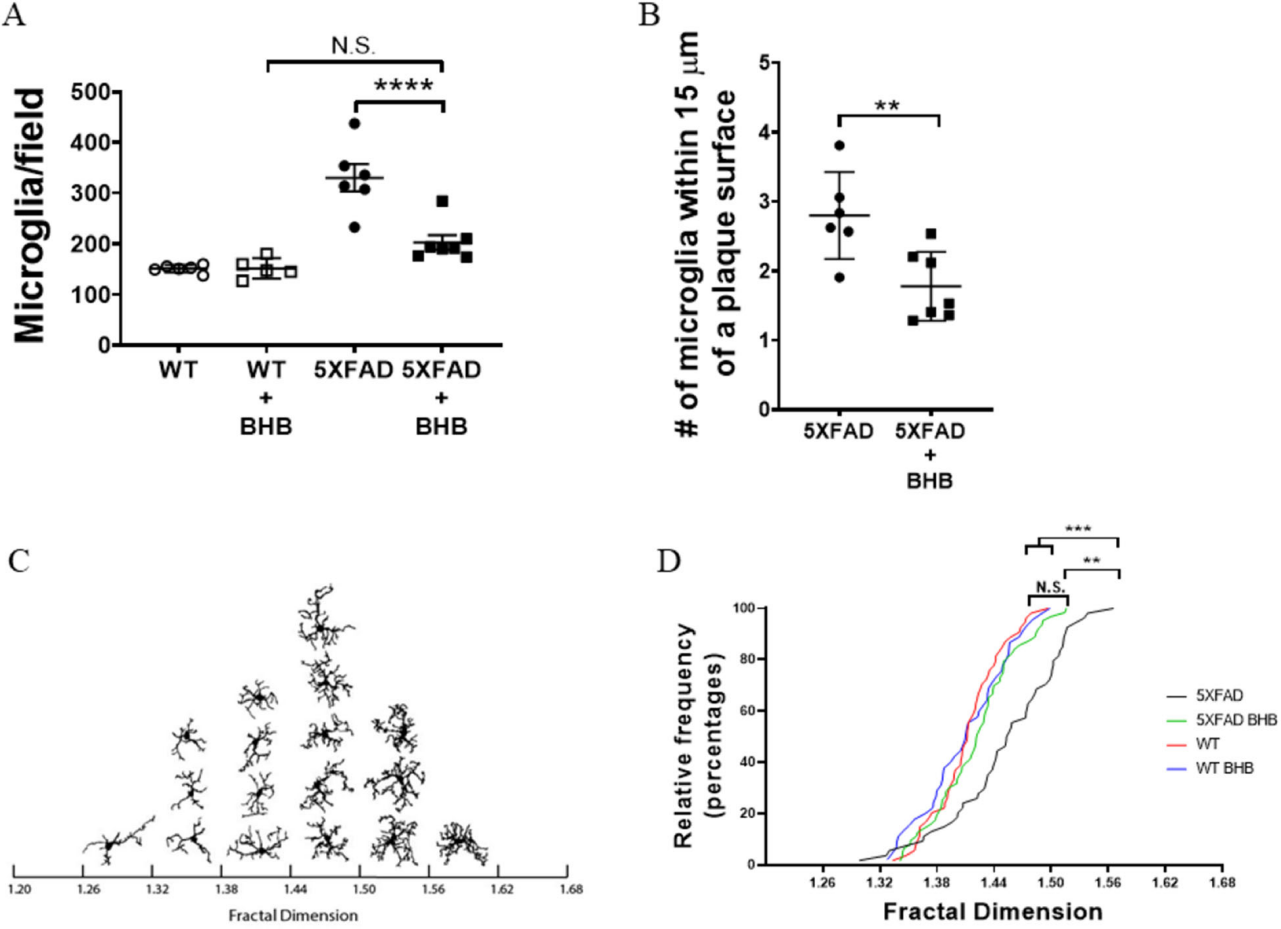
BHB treatment results in less pronounced microgliosis in 5XFAD mice. Brain sections were prepared as in Fig. 2. The number of microglia/field (**a**) and the number of microglia within 15 μm of plaque surfaces (**b**) were determined using Imaris 9.2.1. (**c**) The box-counting method was used to calculate the fractal dimension of outlined versions of non-plaque-associated microglia (*n* = 3/image; 9 microglia/mouse) using FracLac for ImageJ. Microglial cells were outlined and ordered according to least complex (left) to most complex (right). (**d**) Cumulative distribution of fractal dimensions of non-plaque-associated microglia. Graphs show data as the mean ± SD with ****P ≤ 0.0001, ***P ≤ 0.001, **P ≤ 0.01, and N.S. = not significant

Most likely due to decreased plaque-associated neuroinflammation, we observed a significant decrease in microgliosis in the BHB-treated 5XFAD mice when compared with the untreated controls (*P* ≤ 0.0001; one-way ANOVA with a Tukey’s multiple comparison test) (Fig. 3a). Closer examination revealed significantly decreased microglial clustering within 15 μm of plaque surfaces in 5XFAD mice treated with BHB when compared with untreated control mice (*P* = 0.0074; two-tailed Student’s *t* test) (Fig. 3b). The function of plaque-associated microgliosis in the context of AD remains unclear. For example, several groups suggest the microglia accumulation around plaques forms a protective barrier that may limit amyloid toxicity in the adjacent neurons [7, 11, 40]. In contrast, activated microglia release several proinflammatory cytokines (IL-1β, IL-18, TNF-α, IFN-γ, and IL-6) and produce reactive oxygen species, nitric oxide, and many other proinflammatory molecules implicated in neurodegeneration in AD [41]. Furthermore, the study by Sonsa and colleagues suggests microglia may promote the seeding of plaques, as depletion of microglia results in a significant reduction in plaque pathology in a 5XFAD mouse model of AD [42]. Therefore, inhibiting neuroinflammation, especially prior to microgliosis, may be a key attribute of BHB as a therapeutic agent for AD.

Different morphological microglia phenotypes were first described in the human brain in 1919 [43]. Microglia morphology is noticeably modified during disease, suggesting microglia strongly acclimate to local environments [43]. Morphologically, microglia are classified as ramified (resting), activated, and amoeboid (phagocytic) [8]. Broadly, ramified microglia tend to have moderate morphological complexity while amoeboid microglia have low complexity. Upon exposure to prolonged inflammation, activated microglia can enter a primed state where they promote an exaggerated inflammatory response that can contribute to neurodegeneration [44]. These primed microglia adopt a variety of morphologies, but tend to have higher complexity than resting microglia. Morphological changes in microglia have been observed in human AD patients [45] and in mouse models of AD [46–48], yet little is known about how microglial morphology correlates to AD pathology progression. Plaque-associated microglia usually display an amoeboid morphology, but one of the complicating factors in these studies is that microglia can feature more than one activation phenotype [49]. In our study, we specifically looked at morphological complexity in microglia which were not associated with plaques in order to analyze how reducing NLRP3 inflammasome activation alters the amount of proinflammatory microglia on a systemic level. Fractal dimension analysis of microglia morphological complexity showed a distinct difference in the 5XFAD control mice, which took on a complex, primed morphology, when compared with 5XFAD BHB-treated mice (*P* = 0.0068; one-way ANOVA with a Tukey’s multiple comparison test), WT mice (*P* = 0.0002; one-way ANOVA with a Tukey’s multiple comparison test) and WT BHB-treated mice (*P* = 0.0004; one-way ANOVA with a Tukey’s multiple comparison test), with the microglia from BHB-treated 5XFAD mice having a less complex, ramified morphology similar to those of the WT mice (Fig. 3c, d). Additionally, the microglia of BHB-treated 5XFAD mice were similar to the microglia of the WT and WT BHB mice in having a lower density, number of branches, and area than those of the 5XFAD group, further illustrating that BHB treatment shifts microglia away from a primed, proinflammatory morphology toward a less complex, resting morphology (Supplementary Table S1). This analysis showed BHB treatment of 5XFAD caused non-plaque-associated microglia to remain in a ramified, non-activated state, whereas 5XFAD control mice had microglia in a more complex, proinflammatory state. Overall, these data suggest BHB treatment leads to a reduction in the total number of microglia as well as a reduction in the amount of primed, proinflammatory microglia in non-plaque-associated regions, thereby reducing neuroinflammation-associated AD pathology.

### BHB inhibits NLRP3 inflammasome activation

The NLRP3 inflammasome is a critical component of the innate immune system that controls caspase-1 activation and the release of IL-1β and IL-18 in macrophages [50]. We first confirmed that BHB can inhibit NLRP3 inflammasome activation in BMDM, by using the well-characterized NLRP3 activator, ATP [22]. In our hands, treatment of BMDM with 10-mM BHB inhibited NLRP3 activation as shown by significantly reduced levels of IL-1β (*P* ≤ 0.0001; two-way ANOVA with a Tukey’s multiple comparison test) (Supplementary Fig. S1 A) and caspase-1 processing (*P* = 0.0021; two-tailed Student’s *t* test) (Supplementary Fig. S1 B and C). Furthermore, treatment of BMDM with BHB did not alter cell function and viability as shown by similar levels of secretion of the proinflammatory chemokine KC (CXCL1) (Supplementary Fig. S1 D).

**Fig. 4:**
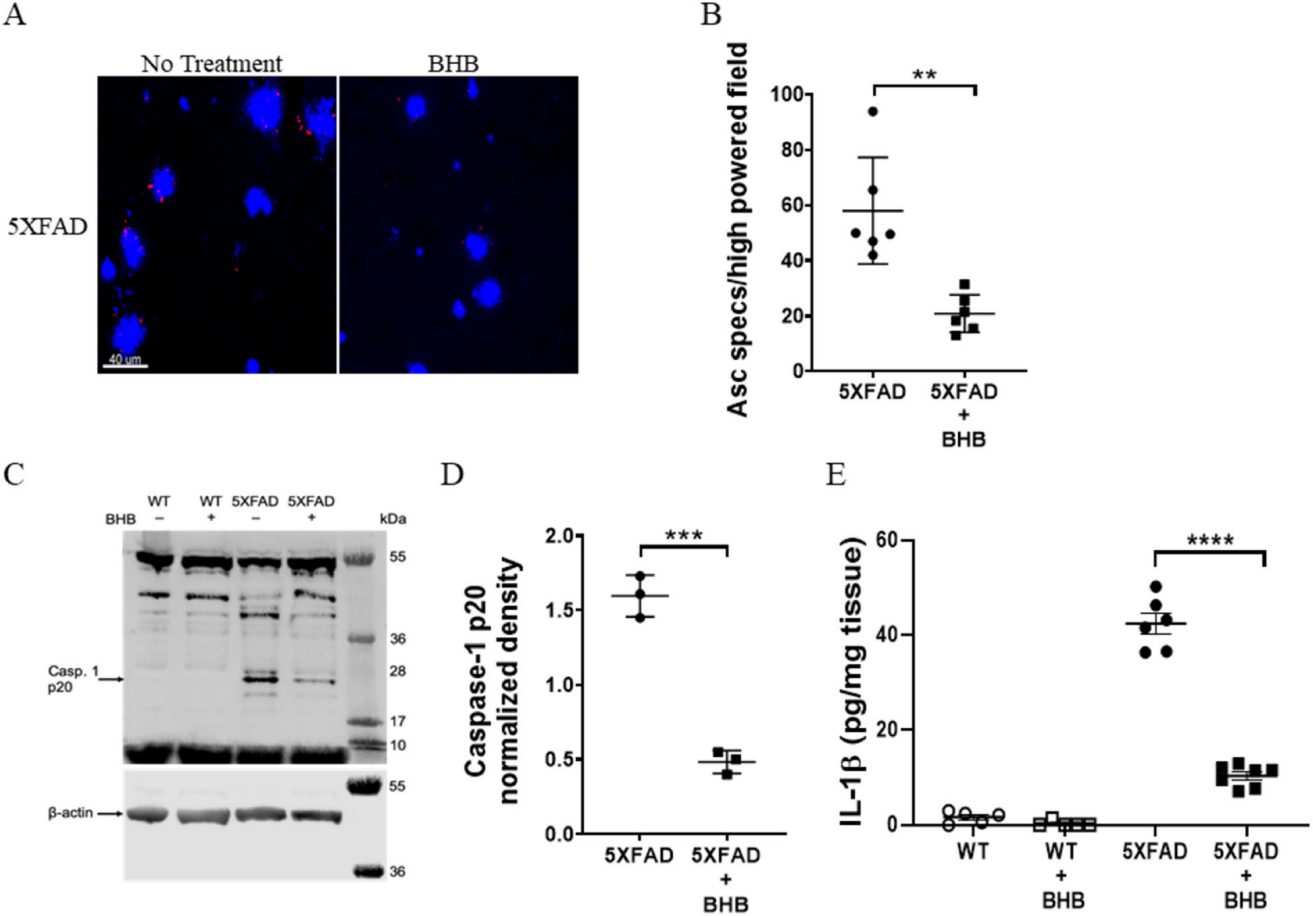
BHB treatment attenuates NLRP3 inflammasome activation in the cortices of 5XFAD mice. Brain sections from 5XFAD mice maintained on regular water (*n* = 6) or BHB containing water (*n* = 6) were stained for Asc specks (red) and plaques (methoxy X04) (blue) (**a**) the number of Asc specks per high powered field in the cortex were determined using Imaris (**b**). Lysates from whole cortices of WT and 5XFAD mice maintained on regular water (*n* = 3/group) or BHB containing water (*n* = 3/group) were analyzed for caspase-1 and β-actin by immunoblot (**c**) and the density of the processed caspase-1 bands were normalized to the β-actin loading control (**d**). Supernatants from cortex homogenates of WT and 5XFAD mice maintained on regular water or BHB-containing water were analyzed for IL-1β by ELISA (**e**). Graphs show data as the mean ± SD with ****P ≤ 0.0001, ***P ≤ 0.001, and **P ≤ 0.01

Consistent with a decrease in inflammasome activation, there were significantly fewer Asc specks in the 5XFAD mice treated with BHB (21.5 specks/HPF) than untreated 5XFAD mice (58 specks/HPF) (*P* = 0.0013; two-tailed Student’s *t* test) (Fig. 4a, b ). Additionally, BHB-treated 5XFAD mice had significantly less processed caspase-1 in their cortices compared with control mice (*P* = 0.0003; two-tailed Student’s *t* test) (Fig. 4c, d). 5XFAD BHB-treated mice also had significantly reduced levels of secreted IL-1β in their cortices compared with control mice (*P* ≤ 0.0001; one-way ANOVA with a Tukey’s multiple comparison test) (Fig. 4e). Additionally, immunoblotting confirmed increased mature IL-1β secretion from the cortices of 5XFAD untreated control mice (Supplementary Fig. S2). Heneka and colleagues demonstrated that NLRP3 inflammasome activation plays a crucial role in Aβ plaque deposition by promoting release of Asc specks by microglia where they bind to Aβ and act as an inflammation-driven component of Aβ pathology [9, 13]. These data suggest inhibition of the NLRP3 inflammasome as a potential mechanism by which BHB attenuates AD pathogenesis. The mechanism by which BHB inhibits NLRP3 inflammasome activation, however, remains unclear. In macrophages, Youm and colleagues suggest that BHB inhibition of the NLRP3 inflammasome occurs independently of G protein-coupled receptor (Gpr109a) and starvation-related mechanisms and that BHB blocks NLRP3 inflammasome activation by regulating upstream events that prevent potassium efflux and reduce Asc speck formation [22]. In primary microglia, Deora and colleagues confirmed BHB inhibited NLRP3 inflammasome activation induced by ATP and monosodium urate (MSU) crystals [51]. Surprisingly, BHB did not inhibit inflammasome activation by synuclein fibrils, suggesting inflammasome activation by synuclein fibrils, and potentially other pathological protein aggregates, occurs independently of the upstream events regulated by BHB described by Youm and colleagues [51]. These findings, however, do not rule out a neuroprotective role for BHB in neurons, which was also demonstrated in a mouse model of Aβ-induced AD-like pathology [31]. While the mechanism by which BHB inhibits NLRP3 inflammasome activation remains unclear, our data suggest that BHB inhibits NLRP3 inflammasome activation to attenuate AD pathology by reduced Asc speck formation, plaque nucleation, and inflammation.

## Conclusions

We found reduced BHB levels in the red blood cells and brain parenchyma of AD patients. A previous study proposed that BHB inhibited NLRP3 inflammasome activation [22]. This study was based on basic research in human and mouse macrophages and suggests BHB as a potential therapeutic agent for NLRP3-mediated chronic inflammatory diseases [22]. Therefore, we tested the potential of BHB to reduce AD pathology by a NLRP3-dependent mechanism in the 5XFAD mouse model of AD. We found that BHB-treated 5XFAD mice had fewer plaques and less pronounced microgliosis than untreated control mice. Importantly, BHB-treated 5XFAD mice had fewer Asc specks and less inflammasome activation in their cortices as indicated by reduced levels of processed caspase-1. Collectively, our data highlight the protective role of BHB-mediated inhibition of the inflammasome in AD. Also, our study suggests increasing BHB levels via dietary regimens or supplementation as a promising preventative and therapeutic strategy for AD.

## Materials and methods

### Human samples

#### Demographics

An autopsy specimen database maintained under the auspices of the Wisconsin Alzheimer’s Disease Research Center was interrogated for the presence of paired brain and blood samples associated with histopathologically documented cases of AD. The diagnosis of AD was made using the NIA-AA criteria [52]. Patients with an NIA-AA score of A3B3C3, (high AD pathologic change) were included in the AD sample group. The database was searched for non-AD control (CTRL) group cases based on histopathological evaluation without pathologic changes of AD. Cases with a diagnosis of “low” or “intermediate” AD change by NIA-AA criteria were excluded. Ten non-AD CTRL cases were selected with the intent of matching gender distribution, age, and postmortem interval between the non-AD CTRL and AD groups (Supplementary Table S2). The primary postmortem histopathologic diagnoses of patients in the two groups is shown in Supplementary Table S3.

#### Brain samples

Fresh frozen 2-g (average) samples of prefrontal cortex were collected at the time of autopsy and stored at − 80 °C until shipped on dry ice to Metabolon (Durham, NC) for untargeted metabolomic analysis. No microdissection of gray and white matters was performed.

#### Blood samples

Whole blood (20 mL) was collected in red-top non-anticoagulated tubes during postmortem examination by cardiac puncture. Samples were aliquoted and stored at − 80 °C until shipped on dry ice to Metabolon (Durham, NC) for untargeted metabolomic analysis. Aliquots from the packed cell volume of samples were obtained for analysis.

#### Sample preparation

Samples were processed using the automated MicroLab® STAR™ system from Hamilton Company. To remove protein and small molecules and to recover chemically diverse metabolites, proteins were precipitated with methanol under vigorous shaking (Glen Mills GenoGrinder 2000) and then were centrifuged. The extract was divided into five fractions. Two fractions were analyzed by two separate reverse phase ultra-performance liquid chromatography tandem mass spectroscopy (RP/UPLC-MS/MS) methods with positive ion mode electrospray ionization (ESI), one was analyzed by RP/UPLC-MS/MS with negative ion mode ESI, one was analyzed by hydrophilic interaction chromatography (HILIC) UPLC-MS/MS with negative ion mode ESI, and one was reserved for backup. Organic solvent was removed by briefly placing samples on a TurboVap (Zymark), and samples were stored under nitrogen overnight prior to analysis.

#### Quality assurance/quality control

Multiple types of controls were analyzed in tandem with experimental samples: a pooled matrix sample generated by taking a small volume of each experimental sample was used as a technical replicate; extracted water samples were used as process blanks; and a cocktail of QC standards that were chosen to not interfere with measurement of endogenous compounds was spiked into every analyzed sample to monitor instrument performance and aid with chromatographic alignment. Instrument variability was calculated using the median relative standard deviation (RSD) for the QC standards added prior to injection. Overall process variability was determined by calculating the median RSD for all endogenous metabolites present in 100% of pooled samples. Experimental samples were randomized across the platform run with QC samples spaced evenly among experimental samples.

#### Mass spectrometry parameters

All analysis utilized a Waters ACQUITY ultra-performance liquid chromatograph (UPLC) and a Thermo Scientific Q-Exactive high-resolution/accurate mass spectrometer, interfaced with a heated electrospray ionization (HESI-II) source and Orbitrap mass analyzer at 35,000 mass resolution. Four methods were used in order to identify as many metabolites as possible.

In the first method, sample aliquots were analyzed using acidic positive ion conditions and were chromatographically optimized for hydrophilic compounds. The extract was eluted from a C18 column (Waters UPLC BEH C18-2.1 × 100 mm, 1.7 μm) using a gradient of water and methanol, containing 0.05% perfluoropentanoic acid (PFPA) and 0.1% formic acid (FA).

In the second method, sample aliquots were analyzed with the same mass spectrometry conditions, but were optimized for hydrophobic compounds. The extract was eluted from the same column mentioned previously using methanol, acetonitrile, and water, with 0.05% PFPA and 0.1% FA. This gradient was operated at an overall higher organic gradient.

A third aliquot was analyzed using basic negative ion mode using a separate C18 column. These extracts were eluted using a gradient of methanol and water with 6.5 mM ammonium bicarbonate at pH 8.

The fourth aliquot was also analyzed via negative ion mode, following an elution from a HILIC column (Waters UPLC BEH Amide 2.1 × 150 mm, 1.7 um) using a gradient of water and acetonitrile with 10 mM ammonium formate at pH 10.8. The scan range varied between methods but covered a range of 70 to 1000 m/z.

#### Identification of features

Raw data was extracted, peak-identified, and QC processed using a LAN backbone and a database server running Oracle 10.2.0.1 Enterprise Edition. These systems are built on a web-service platform utilizing Microsoft’s .NET technologies. Compounds were identified by comparison to a library of purified standards or recurring unknown entities; this library is maintained by Metabolon and contains the retention time/index (RI), mass to charge ratio (m/z), and chromatographic data (including MS/MS spectral data). Biochemical identifications are based on three criteria: retention index within a narrow RI window, accurate mass match to the library ± 10 ppm, and the MS/MS forward and reverse scores between the experimental data and authentic standards. These MS/MS scores are based on a comparison of ions present in the experimental spectrum to ions present in the library spectrum. More than 3300 commercially available purified standard compounds have been acquired and registered into Metabolon’s Laboratory Information Management System (LIMS) for analysis on all platforms. Additional mass spectral entries have been made for structurally unnamed biochemicals, identified due to their recurrent nature.

### Mice

WT (C57BL/6J) and 5XFAD (Tg6799) mice were purchased from Jackson Laboratory. All mice were bred and housed in specific-pathogen-free conditions. At 8 weeks of age, groups of male and female WT and 5XFAD mice were administered BHB (Sigma, Cat. No. H6501)-supplemented water until 16 weeks of age. The target intake of BHB was approximately 3 g/kg of body weight/day. BHB was administered in drinking water at a final concentration of 0.01875 g/ml. The BHB dose was estimated using previous studies which showed effective ketosis induction without causing side effects [22, 53–55]. The Institutional Animal Care and Use Committee at the University of Wisconsin approved all protocols used in this study.

### Preparation of mouse brain samples and confocal microscopy

Brain samples were processed as previously described [3]. Briefly, mice were anesthetized with isoflurane and perfused with ice-cold PBS containing 1 U/ml of heparin. Brains were fixed in 4% PFA for 48 h at 4 °C, rinsed with PBS, and incubated for 48 h at 4 °C in 30% sucrose before freezing in a 2:1 mixture of 30% sucrose and optimal cutting temperature compound. Brain samples were sectioned (40 μm) on a cryostat and stained with anti-Iba1 (Wako, Cat. No. 019-19741) and incubated overnight at 4 °C. Sections were washed with PBS and incubated with anti-rabbit IgG DyLight 549 (Vector Laboratories, Cat. No. DI-1549), methoxy-X04 (Tocris Bioscience, Cat. No. 4920) and TO-PRO-3 (Thermo Fisher Scientific, Cat. No. T3605) for 1 h at room temperature. The methoxy-X04 plaque staining was confirmed by staining with purified anti-β-Amyloid 1–16 (BioLegend, Cat. No. 803001, clone 6E10). Sections were washed and mounted using Fluoromount G (SouthernBiotech, Cat. No. 0100-001). Random images were taken of the cortices immediately dorsal to the hippocampus as previously described [3, 56]. Images were taken using a Nikon A1R confocal microscope at the University of Wisconsin Optical Imaging Core. Images were then processed with Imaris 9.2.1 (Bitplane).

### Image analyses

Quantification of plaque data was performed using the surface feature in Imaris. A complex three-dimensional surface object was created by defining an intensity threshold within the channel containing methoxy-X04. Defined surfaces were inspected to determine if they truly represented plaques. The average volume, sphericity, intensity, and number of these surfaces were calculated in Imaris. The percent of the image volume that these surfaces occupied was calculated by dividing the total volume of the surfaces by the total volume of the image. Two-dimensional maximum intensity projections of these images were exported to ImageJ [57, 58] where a binary threshold was set for the plaques. The percent of the image area that these plaques occupied was calculated using the “Particle Analysis” function.

Analysis of microglial position and localization around plaques was performed in Imaris using scripts from MATLAB (Mathworks). A colocalization channel was created between microglia in the Iba1 channel and the nuclei in the TO-PRO-3 channel. A spots object was then created by defining the intensity threshold within the colocalization channel. The total number of these spots were calculated in Imaris and represented the total number of microglia nuclei in the image. The average number of spots whose center fell within either 15 or 30 μm of the three-dimensional intensity-defined plaque surface were calculated using a modified version of the “Find Spot Close to Surface” MATLAB script.

To quantify the morphological differences in microglia between 5XFAD and WT mice with and without BHB treatment, fractal dimensions were calculated using the ImageJ plug-in FracLac. The fractal dimension is a value that is used to quantify complexity in a binary image by measuring how detail changes with a change in scale. For each mouse, there were three images taken and for each of those images, three microglia were selected (9 microglia analyzed per mouse). The three microglia that were selected were representative of the non-plaque associated microglia in an image. There were three parameters for selecting microglia for analysis: (1) The microglia must be greater than 30 μm away from the nearest plaque (to ensure that the microglia selected were non-plaque associated). (2) The microglia must be clearly distinguishable from its background (to make proper image analysis more accurate). (3) The selected microglia must be representative of the microglia population that meets the previous two criteria (so that abnormally complex or simple microglia are not selected). Microglia from the population that met all three parameters were randomly chosen and further analyzed. Then, two-dimensional max intensity projections of these microglia were exported to ImageJ. A binary threshold was set for each microglia, and background interference was removed from the images. Outlined versions of each microglia were analyzed in FracLac (http://rsbweb.nih.gov/ij/plugins/fraclac/FLHelp/Introduction.htm) using the box-counting method to determine their fractal dimension. The averages of these fractal dimensions were used to compare the morphological differences between mice and their treatment. The “Skeletonize” function in ImageJ was used to obtain data regarding microglia processes characteristics that was also used to quantify morphological changes based on treatment groups.

### Cell culture assays

To prepare BMDM, tibias and femurs were removed from WT (C57BL/6J) mice, and flushed with RPMI 1640 supplemented with Glutamax, non-essential amino acids, sodium pyruvate, penicillin/streptomycin, and 10% heat-inactivated FBS (complete RPMI) and 20% L-cell conditioned medium (LCCM). Cells were plated in petri dishes and cultured for 4–7 days prior to use. For inflammasome activation assays, 5 × 10^5^ BMDM were seeded per well in a 24-well tissue culture plate, primed with LPS (50 ng/ml) from *Escherichia coli* 0111:B4 (Invivogen) for 4 h and stimulated with the NLRP3 activator, ATP (5 mM), for 1.5 h. BHB (Sigma, Cat. No. H6501) (10 mM) was added 4 h prior to inflammasome activation. Following treatment, BMDM were lysed with RIPA buffer (50 mM Tris, 150 mM NaCl, 1% SDS and 1% Triton X-100) containing PMSF, sodium orthovanadate, and Halt™ Protease Inhibitor Single-Use Cocktail (Thermo Fisher Scientific, Cat. No. 78425). Supernatants and lysates were flash frozen on dry ice and stored at − 80 °C until use.

### ELISAs

BMDM supernatants were assayed for IL-1β and KC by ELISA. Antibody pairs for the IL-1β (MAB401 and BAF401; clone 30311 and polyclonal) ELISA were purchased from R&D Systems. The KC ELISA was performed using the Mouse CXCL1/KC DuoSet ELISA (R&D Systems, Cat. No. DY453) according to the manufacturer’s instructions.

For the BHB ELISA, 10 mg of cortex was removed from the brains of mice. The tissue was disrupted, and the proteins were precipitated with perchloric acid. The samples were centrifuged, and the cleared supernatants were analyzed using the colorimetric β-hydroxybutyrate Assay Kit (Abcam, Cat. No. ab83390) according to the manufacturer’s instructions.

Supernatants from mouse brain cortex homogenates were assayed for IL-1β by ELISA as described above. The supernatants were prepared by homogenizing 2.5 mg of cortex in 0.25 ml of sterile PBS. Cells and debris were removed by centrifugation, and supernatants were collected for analysis.

### Immunoblotting

BMDM supernatants, BMDM lysates, whole cortex brain lysates, and PBS soluble brain supernatants were prepared by adding NuPAGE LDS sample buffer (Invitrogen) and 10% β-mercaptoethanol and boiling for 10 min at 85 °C. Samples were run on NuPAGE 4-12% Bis–Tris precast gels (Invitrogen), transferred to nitrocellulose membranes, and blocked with 5% milk in PBS plus 0.05% Tween 20 (PBST) for 1 h at room temperature. Membranes were probed with anti-Caspase-1 (p20) (mouse), mAb (Casper-1) (Adipogen, Cat. No. AG-20B-0042) or anti-IL-1β (Abcam, Cat. No. ab234437) in 5% milk in PBST overnight at 4 °C. Membranes were subsequently washed and incubated with IR Dye® 800 CW goat anti-mouse antibody (LI-COR, Cat. No. 926-32210) for 2 h at room temperature, washed, and scanned with the Odyssey CLx (LI-COR) imaging system. For lysates, membranes were washed and stripped using Restore™ Western Blot Stripping Buffer (Thermo Fisher Scientific). Membranes were subsequently reprobed with β-actin antibody (Cell Signaling Technology, Cat. No. 4970), incubated with IR Dye® 800 CW goat anti-rabbit antibody (LI-COR, Cat. No. 926-32211) and imaged as described above. The densities of the caspase-1 bands were normalized to the β-actin loading control using Fiji/ImageJ software [57, 58].

### Statistical analyses

Statistical analysis was performed using Prism 8.3.1 (GraphPad). Data in figures are presented as mean ± SEM, or mean ± SD, as indicated in the figure legends. Quantification of immunoblots and confocal microscopy images were performed using Imaris, MATLAB, Fiji and ImageJ. A two-tailed Student’s *t* test was used to compare one variable across two conditions. For experiments in which one variable was compared in more than two conditions, one-way ANOVA with a Tukey’s multiple comparison test was used. Two-way ANOVA with a Tukey’s multiple comparison test was used to compare two variables across multiple conditions. For the fractal dimension analysis, a Kruskal–Wallis test with a Dunn’s multiple comparison test was used to determine differences amongst each group. A *P* value ≤ 0.05 (*P ≤ 0.05, **P ≤ 0.01, ***P ≤ 0.001, and ****P ≤ 0.0001) was used as the significance cutoff.

## Supplementary information

**Additional file 1: Figure S1.** BHB inhibits NLRP3 inflammasome activation in BMDM. **Fig. S2.** BHB treatment reduces mature IL-1β secretion in the cortices of 5XFAD mice. **Table S1.** Summary of microglial morphology data. **Table S2.** Demographics data of AD versus Non-AD CTRL. **Table S3.** Primary neuropathologic diagnosis in AD versus Non-AD CTRL

## Data Availability

All data generated or analyzed during this study are included in this published article (and its supplementary information files). Ethics approval and consent to participate The Institutional Animal Care and Use Committee at the University of Wisconsin approved all protocols used in this study.

## Abbreviations

Aβ: Amyloid-β
AD: Alzheimer’s disease
Asc: Apoptosis-associated speck-like protein containing a caspase recruitment domain
BHB: β-Hydroxybutyrate
BMDM: Bone marrow-derived macrophages
NLRP3: Nod-like receptor family pyrin domain containing 3
WT: Wild-type
