# Supplementary material for: β-Hydroxybutyrate inhibits inflammasome activation to attenuate Alzheimer’s disease pathology
Source: J Neuroinflammation. 2020 Sep 21;17:280. doi: 10.1186/s12974-020-01948-5 (PMC7507727; doi:10.1186/s12974-020-01948-5)
Supplement: Supplementary file 1 — Additional file 1: Figure S1. BHB inhibits NLRP3 inflammasome activation in BMDM. Fig. S2. BHB treatment reduces mature IL-1β secretion in the cortices of 5XFAD mice. Table S1. Summary of microglial morphology data. Table S2. Demographics data of AD versus Non-AD CTRL. Table S3. Primary neuropathologic diagnosis in AD versus Non-AD CTRL [file 12974_2020_1948_MOESM1_ESM.docx]

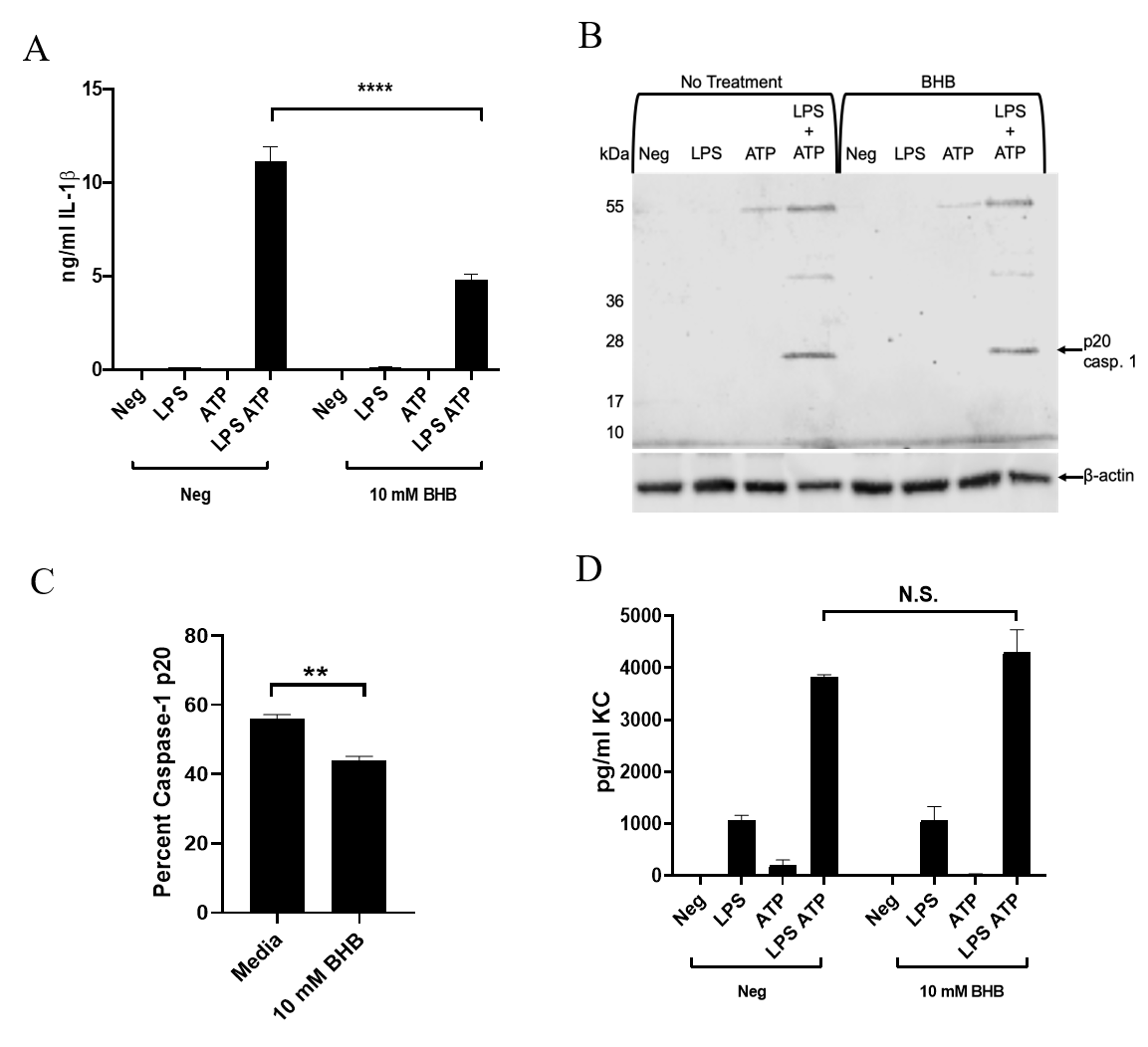


**Figure S1.** **BHB inhibits NLRP3 inflammasome activation in BMDM.** BMDM were primed with LPS (50 ng/ml) for 4 h and stimulated with ATP (5 mM) for 1.5 h. Supernatants were assessed for IL-1β by ELISA **(A)** immunoblot for caspase-1 **(B)** caspase-1 p20 bands were quantified and normalized to the β-actin loading control **(C)** and secreted KC by ELISA (**D)**. Experiments were performed in triplicate. Results are representative of 5 independent experiments. Data are shown as the mean ± SEM. *****P* ≤ 0.0001, ***P* ≤ 0.01, N.S. = not significant.


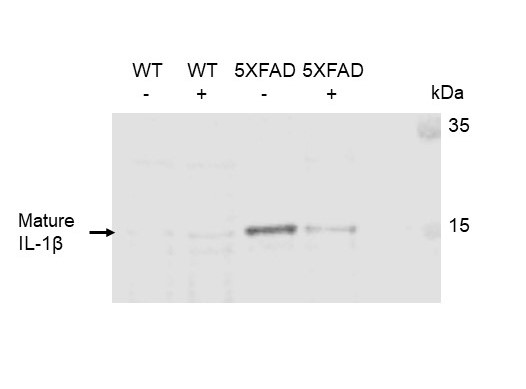


**Fig. S2. BHB treatment reduces mature IL-1β secretion in the cortices of 5XFAD mice.** Supernatants from cortex homogenates of WT and 5XFAD mice maintained on regular water or BHB containing water were analyzed for mature IL-1β by immunoblot. Results are representative of three independent experiments.

**Table S1.** **Summary of microglial morphology data**

| Group | Density | Fractal Dimension | Number of Branches | Area (µm²) |
| --- | --- | --- | --- | --- |
| WT | 11.02 | 1.41 | 138.50 | 203.48 |
| WT BHB | 11.80 | 1.41 | 120.78 | 189.73 |
| 5XFAD | 13.23 | 1.45 | 177.37 | 262.03 |
| 5XFAD BHB | 12.07 | 1.42 | 143.32 | 224.95 |

BHB, β-hydroxybutyrate; WT, wild-type

**Table S2. Demographics data of AD versus Non-AD CTRL**

|  |  | Non-AD CTRL  (n = 10) |  | AD  (n = 10) |  | *P* value |
| --- | --- | --- | --- | --- | --- | --- |
| Age (mean) |  | 79 |  | 81 |  | 0.76 |
| Sex M:F |  | 1:4 |  | 1:4 |  | N/A |
| PMI, hours:minutes (mean) |  | 7:58 |  | 8:05 |  | 0.98 |

AD, Alzheimer’s disease; CTRL, control; PMI, post-mortem interval

**Table S3. Primary neuropathologic diagnosis in AD versus Non-AD CTRL**

| Non-AD CTRL  (n = 10) | Age  (Years) | Sex | PMI  (hours:  minutes) |  |  | Diagnosis |  |  | AD  (n = 10) | Age  (Years) | Sex | PMI  (hours:  minutes) |  |  | Diagnosis |
| --- | --- | --- | --- | --- | --- | --- | --- | --- | --- | --- | --- | --- | --- | --- | --- |
| Non-AD CTRL 1 | 86 | F | 3:30 |  |  | Hippocampal sclerosis  A0B0C0 |  |  | AD 1 | 63 | F | 3:18 |  |  | Alzheimer’s disease  A3B3C3 |
| Non-AD CTRL 2 | 41 | F | 4:15 |  |  | Leukodystrophy  A0B0C0 |  |  | AD 2 | 89 | F | 3:22 |  |  | Alzheimer’s disease  A3B3C3 |
| Non-AD CTRL 3 | 94 | M | 5:18 |  |  | Argyrophilic grain disease  A0B0C0 |  |  | AD 3 | 89 | F | 4:15 |  |  | Alzheimer’s disease  A3B3C3 |
| Non-AD CTRL 4 | 86 | F | 6:22 |  |  | Glioblastoma  A0B0C0 |  |  | AD 4 | 86 | F | 4:25 |  |  | Alzheimer’s disease  A3B3C3 |
| Non-AD CTRL 5 | 86 | F | 11:40 |  |  | Hippocampal sclerosis  A0B0C0 |  |  | AD 5 | 72 | F | 4:26 |  |  | Alzheimer’s disease  A3B3C3 |
| Non-AD CTRL 6 | 95 | M | 15:24 |  |  | Normal adult brain  A0B0C0 |  |  | AD 6 | 79 | M | 5:18 |  |  | Alzheimer’s disease  A3B3C3 |
| Non-AD CTRL 7 | 70 | F | 5:05 |  |  | Normal adult brain  A0B0C0 |  |  | AD 7 | 73 | F | 9:10 |  |  | Alzheimer’s disease  A3B3C3 |
| Non-AD CTRL 8 | 80 | F | 3:00 |  |  | Parkinson plus  A0B0C0 |  |  | AD 8 | 87 | F | 11:45 |  |  | Alzheimer’s disease  A3B3C3 |
| Non-AD CTRL 9 | 69 | F | 6:00 |  |  | Amyotrophic lateral sclerosis  A0B0C0 |  |  | AD 9 | 80 | M | 15:25 |  |  | Alzheimer’s disease  A3B3C3 |
| Non-AD CTRL 10 | 81 | F | 19:15 |  |  | Hippocampal sclerosis  A0B0C0 |  |  | AD 10 | 88 | F | 19:30 |  |  | Alzheimer’s disease  A3B3C3 |

AD, Alzheimer’s disease; CTRL, control; PMI, post-mortem interval; A0B0C0, no clinical or histopathological evidence of AD; A3B3C3, high probability of AD based on NIA-AA criteria
